# TGFβR signalling controls CD103^+^CD11b^+^ dendritic cell development in the intestine

**DOI:** 10.1038/s41467-017-00658-6

**Published:** 2017-09-20

**Authors:** C. C. Bain, J. Montgomery, C. L. Scott, J. M. Kel, M. J. H. Girard-Madoux, L. Martens, T. F. P. Zangerle-Murray, J. Ober-Blöbaum, D. Lindenbergh-Kortleve, J. N. Samsom, S. Henri, T. Lawrence, Y. Saeys, B. Malissen, M. Dalod, B. E. Clausen, A. McI. Mowat

**Affiliations:** 1 Centre for Immunobiology, Institute of Infection, Immunity and Inflammation, College of Medicine, Veterinary Medicine and Life Sciences, University of Glasgow, Glasgow, G12 8TJ UK; 2 Laboratory of Myeloid Cell Ontogeny and Functional Specialization, VIB-UGent Center for Inflammation Research, Technologiepark, Ghent 927, Belgium; 3 Department of Biomedical Molecular Biology, Ghent University, Ghent, 9000 Belgium; 4 Department of Immunology, Erasmus MC, University Medical Center, 3015 GE Rotterdam, The Netherlands; 5 Data Mining and Modeling for Biomedicine, VIB Inflammation Research Center, Ghent, 9052 Belgium; 6 Institute for Molecular Medicine, University Medical Centre of the Johannes Gutenberg University, 55131 Mainz, Germany; 7 Laboratory of Pediatrics, Division of Gastroenterology and Nutrition, Erasmus Medical Center, Rotterdam, 3015 GE The Netherlands; 8 Centre d’Immunologie de Marseille-Luminy, Aix Marseille Université UM2, INSERM, U1104, CNRS UMR7280, 13288 Marseille, France; 9 Department of Applied Mathematics, Computer Science and Statistics, Ghent University, Ghent, 9000 Belgium; 10 Present Address: The University of Edinburgh/MRC Centre for Inflammation Research, University of Edinburgh, Edinburgh, EH16 4TJ UK; 11 Present Address: Faculty of Biology, Medicine and Health and Manchester Collaborative Centre for Inflammation Research, University of Manchester, Manchester, M13 9PT UK

## Abstract

CD103^+^CD11b^+^ dendritic cells (DCs) are unique to the intestine, but the factors governing their differentiation are unclear. Here we show that transforming growth factor receptor 1 (TGFβR1) has an indispensable, cell intrinsic role in the development of these cells. Deletion of *Tgfbr1* results in markedly fewer intestinal CD103^+^CD11b^+^ DCs and a reciprocal increase in the CD103^−^CD11b^+^ dendritic cell subset. Transcriptional profiling identifies markers that define the CD103^+^CD11b^+^ DC lineage, including CD101, TREM1 and Siglec-F, and shows that the absence of CD103^+^CD11b^+^ DCs in CD11c*-*Cre*.Tgfbr1* ^fl/fl^ mice reflects defective differentiation from CD103^−^CD11b^+^ intermediaries, rather than an isolated loss of CD103 expression. The defect in CD103^+^CD11b^+^ DCs is accompanied by reduced generation of antigen-specific, inducible FoxP3^+^ regulatory T cells in vitro and in vivo, and by reduced numbers of endogenous Th17 cells in the intestinal mucosa. Thus, TGFβR1-mediated signalling may explain the tissue-specific development of these unique DCs.

## Abstract (web summary)

Developmental cues for the different dendritic cell (DC) subsets in the intestine are yet to be defined. Here the authors show that TGFβR1 signalling is needed for development of CD103^+^CD11b^+^ intestinal DCs from CD103^−^CD11b^+^ cells and that they contribute to the generation of Th17 and regulatory T cells

## Introduction

Dendritic cells (DCs) are central to the regulation of immune function in the intestine. They control whether tolerance or active immunity is induced by different kinds of antigens, specify the nature of responses that occur and imprint primed T and B cells with the selective ability to return to the intestinal mucosa^1^. Although most of these events take place when DCs encounter naive lymphocytes in the draining mesenteric and colonic lymph nodes^2–4^, the relevant DCs are derived from the Peyer’s patches (PPs) or lamina propria (LP) of the mucosa itself, where they capture antigen before emigrating to lymph nodes in afferent lymphatics^5^. Therefore, exploring the biology of mucosal DCs is essential to understand the cellular basis of immune responses in the intestine.

Work by ourselves and others has revealed heterogeneity among DCs in the mouse intestine, with four major subsets based on the expression of CD103 and CD11b^6–8^. These cells include a prominent population of CD103^+^CD11b^+^ DCs, a subset unique to the intestine and its draining lymphoid tissues^5^. Intestinal CD103^+^CD11b^+^ DCs are functionally and ontogenically distinct from the Batf3/IRF8-dependent, XCR1^+^CD103^+^CD11b^−^ DCs with cross-presenting activity in the gut and elsewhere^9–11^, commonly referred to as conventional DC subset 1 (cDC1)^12^. However, the development of CD103^+^CD11b^+^ DCs is yet to be dissected fully. Several factors have been reported to be important for the homeostasis of these cells including colony-stimulating factor 2 (CSF2)^13^, Notch-2^14^, IRF4^8, 15^, retinoic acid^16^ and signal regulatory protein α (SIRPα)^17^. However, whether these factors link to a common developmental pathway and why this unusual population is restricted to the intestine are unclear.

Transforming growth factor β (TGFβ) is abundant in the intestine and induces the expression of CD103 on intestinal intraepithelial lymphocytes and regulatory T (Treg) cells with effector function^18, 19^. TGFβ is also reported to influence the development and/or homeostasis of several myeloid cell populations, including Langerhans cells and microglia^20–23^. However, the role of TGFβ-mediated signalling in DC development in the intestinal mucosa has not been addressed directly. Using refined approaches we have developed to identify DC subsets and other CD11c^+^ cells in the intestine^6, 24^, we show here that mice lacking the TGFβR1 on CD11c^+^ cells have a selective and cell-intrinsic defect in CD103^+^CD11b^+^ DCs in the intestine. By developing a panel of markers that defines this lineage, we show that the loss of CD103^+^CD11b^+^ DCs reflects a defect in differentiation from a CD103^−^CD11b^+^ intermediate, rather than just the absence of CD103 expression. The lack of TGFβR1-dependent CD103^+^CD11b^+^ DCs is accompanied by defective generation of antigen-specific, inducible FoxP3^+^ Treg cells in vitro and in vivo, and by reduced numbers of Th17 cells in the intestinal mucosa. Thus, TGFβ-mediated signalling is indispensable for the phenotypic and functional imprinting of LP CD103^+^CD11b^+^ DCs.

## Results

### T-cell-dependent inflammation in CD11c-Cre.*Tgfbr1*^fl/fl^ mice

**Fig. 1:**
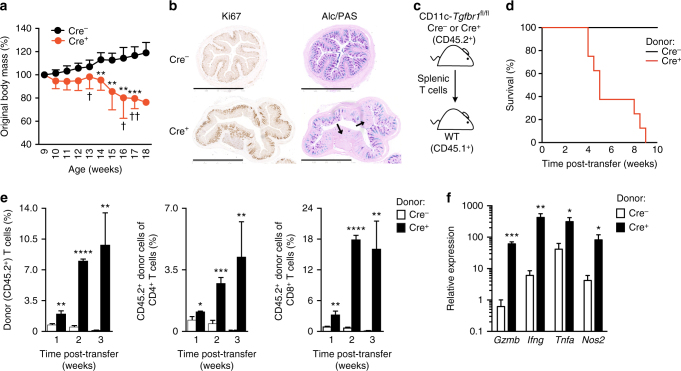
T-cell-dependent inflammatory disease in CD11c-Cre.*Tgfbr1* ^fl/fl^ mice. **a** Body weights of CD11c*-*Cre.*Tgfbr1* ^fl/fl^ (Cre^+^) mice and *Tgfbr1* ^fl/fl^ (Cre^−^) mice littermate controls presented as a percentage of original bodyweight at 9 weeks of age. The results are the means ± 1 SD of five (Cre^+^) or six (Cre^−^) mice per group and are representative of two experiments (**p < 0.01 and ***p < 0.001—Student’s *t*-test followed by Holm–Sidak correction). († indicates the loss of animal from group). **b** Representative colonic pathology as assessed by epithelial cell turnover (Ki67 staining—*left panels*) and goblet cell density (PAS staining—*right panels*) in Cre^−^ (*upper panels*) and Cre^+^ mice (*lower panels*). *Arrows* indicate loss of goblet cells. *Scale bar*, 1 mm. **c** Experimental scheme for the transfer of splenic T cells from Cre^−^ or Cre^*+*^ mice into congenic WT recipients. **d** Survival of WT recipients given T cells from Cre^−^ ro Cre^*+*^ mice. Data are pooled from two experiments with a total of seven (Cre^−^) or eight (Cre^*+*^) mice per group. **e** Frequency of CD45.2^+^ donor T cells among total blood T cells (*left*) and the frequency of donor cells within the CD4^+^ and CD8^+^ T-cell compartments (*centre* and *right*) at 1, 2 and 3 weeks post transfer in the mice from **c**, **d** above. Data are from one of two independent experiments each with 3 (Cre^*+*^) or 4 (Cre^−^) mice per group. **f** Expression of mRNA transcripts for *Gzmb*, *Ifng*, *Tnfa* and *Nos2* in the stomach of recipients of splenic T cells from Cre^−^ or Cre^*+*^ mice in **d** above. In **e**, **f**, *bars* represent the mean + SD of three mice per group and mRNA expression is relative to expression of *Gapdh*. *p < 0.05, **p < 0.01, ***p < 0.001 and ****p < 0.0005 determined by two-tailed Student’s *t*-test

To explore the role of TGFβ in intestinal DC development, we crossed *Tgfbr1* ^fl/fl^ mice with *Itgax*-Cre mice, which constitutively express Cre recombinase under control of the CD11c promoter^25^ (referred to here as CD11c*-*Cre). Although CD11c*-*Cre*.Tgfbr1* ^fl/fl^ mice (Cre^+^) and non-transgenic *Tgfbr1* ^fl/fl^ littermates (Cre^−^) were born at Mendelian frequencies, Cre^+^ mice developed a wasting disease and died before 20 weeks of age (Figs. 1a, b and Supplementary Fig. 1a). This was associated with activation of splenic T cells, inflammation of the stomach, colon, liver and lungs, where there were leukocytic infiltrates and the production of pro-inflammatory cytokines (Supplementary Fig. 1b–f). These findings are consistent with a previous report of wasting disease in CD11c*-*Cre*-Tgfbr2* ^fl/fl^ mice^26^ and highlight the need for TGFβ signalling in control of inflammatory pathology.

As some T cells may exhibit functional Cre activity in CD11c-Cre mice^25^ and disruption of TGFβ signalling in T cells is known to provoke a lethal inflammatory disease^27, 28^, we examined whether lack of TGFβR1 signalling in T cells might contribute to the inflammatory disease in CD11c*-*Cre*.Tgfbr1* ^fl/fl^ mice. Analysis of T cells purified from the spleen of these mice demonstrated clear Cre-mediated deletion of genomic *Tgfbr1* (Supplementary Fig. 1g), whereas ~ 8% of circulating T cells and ~ 12% of CD3^+^ small intestinal LP (SILP) T cells were labelled in CD11c*-*Cre*.Rosa26*-LSL-YFP mice (Supplementary Fig. 2a, b). Thus, Cre-mediated recombination occurs in T cells in the CD11c-Cre strain. Furthermore, CD3^+^ T cells from Cre^+^ mice transferred the lethal wasting disease into congenic wild-type (WT) recipients, with expansion and activation of donor CD4^+^ and CD8^+^ T cells, gastritis and expression of messenger RNA for proinflammatory mediators in the stomach (Figs. 1c–f). In contrast, T cells from Cre^−^ littermates showed only limited and transient expansion in recipient mice and these animals remained healthy. Of note, a significant fraction of T cells transferred from Cre^+^ donors had lost the *Tgfbr1* gene (Supplementary Fig. 1g) and crossing the CD11c*-*Cre*.Tgfbr1* ^fl/fl^ mice on to the *Rag1* ^−*/*−^ background prevented wasting disease, confirming that the pathology involved deletion of the TGFβR1 in T cells.

### CD11c^+^ myeloid cells prevent T-cell-mediated colitis

**Fig. 2:**
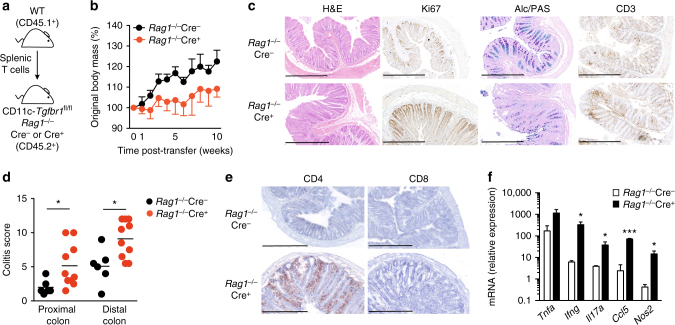
TGFβR1 signalling in CD11c^+^ myeloid cells prevents T-cell-mediated colitis. **a** Experimental scheme for induction of colitis by transfer of CD45.1^+^ WT spleen T cells mice into *Rag1* ^−*/*−^ *Tgfbr1* ^fl/fl^ (*Rag1* ^−*/*−^Cre^−^) or *Rag1* ^−*/*−^ CD11c*-*Cre.*Tgfbr1* ^fl/fl^ (*Rag1* ^−*/*−^Cre^+^) recipients. **b** Body weights following adoptive transfer of WT T cells shown as percentage of starting weight. The results are the means ± 1 SD of four mice per group from one of two independent experiments. **c** Representative colonic pathology as assessed by haematoxylin and eosin staining, Ki67 staining of dividing epithelial cells, PAS^+^ goblet cells and CD3^+^ T cells at 10–17 weeks post-transfer. *Scale bar*, 500 μm. **d** Histological scoring of proximal and distal colon. Data are pooled from four independent experiments with a total of six (*Rag1* ^−*/*−^Cre^−^) or nine (*Rag1* ^−*/*−^Cre^*+*^) recipients per group. *p < 0.05 determined by Mann–Whitney test. **e** CD4 and CD8 expression in colon at 10–17 weeks post transfer. *Scale bar*, 500 μm. **f** Expression of mRNA transcripts for *Tnfa*, *Ifng*, *Il17a*, *Ccl5* and *Nos2* in the colon. *Error bars* represent the mean + 1 SD of two (*Rag1* ^−*/*−^Cre^−^) or five (*Rag1* ^−*/*−^Cre^*+*^) mice per group and mRNA expression is relative to expression of *Abl*. Data are from one of three independent experiments performed. *p < 0.05 and ***p < 0.001 determined by two-tailed Student’s *t*-test

Although these findings implied that TGFβR1 signalling in T cells normally prevents inflammatory disease, previous work suggested that the wasting disease seen in CD11c*-*Cre*-Tgfbr2* ^fl/fl^ mice was dependent on DCs^26^. To investigate whether this might also contribute to the development of inflammatory disease in CD11c*-*Cre*.Tgfbr1* ^fl/fl^ mice, we transferred total T cells from WT (CD45.1) donors into *Rag1* ^−*/*−^ CD11c*-*Cre*.Tgfbr1* ^fl/fl^ mice (hereafter referred to as *Rag1* ^−*/*−^ Cre^+^) or *Rag1* ^−*/*−^ *Tgfbr1* ^fl/fl^ mice (hereafter referred to as *Rag1* ^−*/*−^ Cre^−^) (Fig. 2a). Strikingly, following T-cell transfer, *Rag1* ^−*/*−^Cre^+^ recipient mice showed marked growth retardation compared with *Rag1* ^−*/*−^Cre^−^ control mice (Fig. 2b), together with severe colitis associated with accumulation of CD4^+^ T cells and expression of mRNA for tumour necrosis factor-α, inducible nitric oxide synthase, interferon (IFN)-γ and interleukin (IL)-17A (Figs. 2b–f). Thus, the absence of TGFβR1-mediated signalling in CD11c-expressing cells other than T cells also contributes to T-cell-driven colitis in CD11c*-*Cre*.Tgfbr1* ^fl/fl^ mice.

### TGFβR1 signalling controls intestinal DC homeostasis

**Fig. 3:**
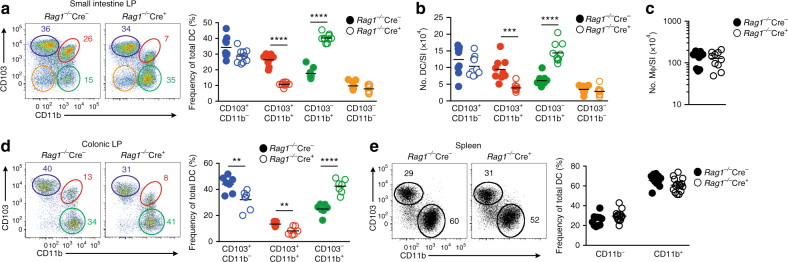
TGFβR1 signalling controls dendritic cell homeostasis in the intestine. **a** Expression of CD103 and CD11b by live CD45^+^CD11c^+^MHCII^+^CD64^−^ DC (*left*) and relative frequencies of DC subsets (*right*) in the SILP of *Rag1* ^−*/*−^ *Tgfbr1* ^fl/fl^ mice (*Rag1* ^−*/*−^Cre^−^) or *Rag1* ^−*/*−^ CD11c-Cre.*Tgfbr1* ^fl/fl^ (*Rag1* ^−*/*−^Cre^+^) mice. **b** Absolute numbers of DC subsets and **c** CD64^+^Ly6C^−^ macrophages from SI from mice in **a**. Data are pooled from three independent experiments with 9 (*Rag1* ^−*/*−^Cre^−^) or 10 (*Rag1* ^−*/*−^Cre^*+*^) mice per group. **d** Expression of CD103 and CD11b by live CD45^+^CD11c^+^MHCII^+^CD64^−^ DC (*left*) and relative frequencies of DC subsets (*right*) in the colonic LP of *Rag1* ^−*/*−^Cre^−^ or *Rag1* ^−*/*−^Cre^+^ mice. Data are pooled from two independent experiments with a total of seven mice per group. **e** Expression of CD103 and CD11b by live CD11c^+^MHCII^+^ DC (*left*) and relative frequencies of DC subsets (*right*) in the spleen of *Rag1* ^−*/*−^Cre^−^ or *Rag1* ^−*/*−^Cre^+^ mice. Data are pooled from four independent experiments with 11 (*Rag1* ^−*/*−^Cre^−^) or 15 (*Rag1* ^−*/*−^Cre^*+*^) mice/group. *p < 0.05, **p < 0.01, ***p < 0.001 and ****p < 0.0001 determined by Student’s *t*-test followed by Holm–Sidak correction

We reasoned that this inflammation may reflect a role for the mononuclear phagocytes that contribute to the generation and/or differentiation of pathogenic T cells in the intestine and thus we explored the populations of DCs and macrophages present in the mucosa of *Rag1* ^−*/*−^Cre^+^ mice. As has been found in immunocompetent mice^6, 7, 29^, the SILP of *Rag1* ^−*/*−^Cre^−^ mice contained four subsets of bona fide CD11c^+^MHCII^+^CD64^−^ DCs, distinguished by their differential expression of CD103 and CD11b (Fig. 3a and Supplementary Fig. 2a). The absolute numbers and proportions of DCs co-expressing CD11b and CD103 were severely reduced in *Rag1* ^−*/*−^Cre^+^ mice compared with *Rag1* ^−*/*−^Cre^−^ or Cre^*+*^ *Tgfbr1* ^*fl/+*^ littermate controls, with parallel increases in the CD11b^+^CD103^−^ DC subset (Figs. 3a, b and Supplementary Fig. 2b). CD103^+^CD11b^−^ and CD103^−^CD11b^−^ DCs were present at comparable frequencies and numbers in *Rag1* ^−*/*−^Cre^+^ and Cre^−^ mice, although as with the remaining CD103^+^CD11b^+^ DCs, CD103^+^CD11b^−^ DCs showed reduced levels of CD103 expression in Cre^+^ mice (Figs. 3a, b). As the small CD103^−^CD11b^−^ subset is variable in size, is phenotypically heterogeneous and may derive from isolated lymphoid follicles^7^, it has not been ascribed specific functions in the LP and it is not explored further here. Importantly, despite expressing high levels of CD11c and exhibiting Cre activity (Supplementary Fig. 3a), the absolute numbers of CD64^+^ mϕ were equivalent in the small intestine of *Rag1* ^−*/*−^Cre^+^ and *Rag1* ^−*/*−^Cre^−^ mice (Fig. 3c). There was a similar reduction in CD103^+^CD11b^+^ DCs in the colonic mucosa of *Rag1* ^−*/*−^Cre^+^ mice compared with *Rag1* ^−*/*−^Cre^−^ littermate controls, together with an increased proportion of the CD103^−^CD11b^+^ subset. Unlike the small intestine, the frequencies of CD103^+^CD11b^−^ DCs were also reduced in the colon of *Rag1* ^−*/*−^Cre^+^ mice (Fig. 3d). Notably, there were no differences in the populations of CD11b^+^ and CD11b^−^ DCs in the spleen of *Rag1* ^−*/*−^Cre^+^ and *Rag1* ^−*/*−^Cre^−^ mice (Fig. 3e). We were unable to obtain sufficient cells from the hypotrophic lymph nodes of *Rag1* ^−*/*−^ mice to assess DC populations in these tissues.

Thus, CD11c-driven deletion of the TGFβR1 leads to a dramatic reduction in the number of CD103^+^CD11b^+^ DCs in the intestinal mucosa. To explore the underlying mechanism in more detail, we focused on the small intestine, where the relevant DC subset is most abundant.

### TGFβR1-mediated control of DC homeostasis is cell intrinsic

**Fig. 4:**
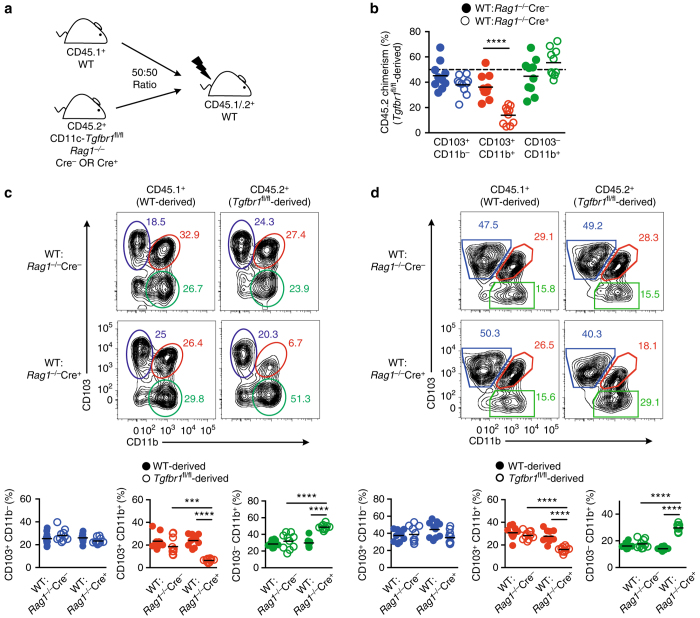
TGFβR1-mediated control of DC homeostasis is cell intrinsic. **a** Experimental scheme for generation of mixed BM chimeric mice by reconstitution of irradiated CD45.1^+^ × CD45.2^+^ mice with a 1 : 1 mixture of BM from CD45.1^+^ WT and *Rag1* ^−*/*−^Cre^−^ or *Rag1* ^−*/*−^Cre^*+*^ CD45.2^+^ donors. **b** CD45.2^+^ *Tgfbr1* ^fl/fl^-derived chimerism among DC subsets from WT:*Rag1* ^−*/*−^Cre^−^ or WT: *Rag1* ^−*/*−^Cre^*+*^ mixed BM chimeras 8–12 weeks post reconstitution. Data are pooled from two independent experiments with a total of 10 mice per group and dotted line represents input chimerism. ****p < 0.0001 determined by Student’s *t*-test followed by Holm–Sidak correction. **c**, **d** Representative expression of CD103 and CD11b by CD45.1^+^ (WT-derived) or CD45.2^+^ (*Tgfbr1* ^fl/fl^-derived) CD11c^+^MHCII^+^CD64^−^ cells from the SILP **c** or MLN **d** of WT:*Rag1* ^−*/*−^Cre^−^ or WT:*Rag1* ^−*/*−^Cre^*+*^ mixed BM chimeras 8–12 weeks post reconstitution. Scatter plots show the frequency of each DC subset of the total DC pool derived from each BM source. Data are pooled from two independent experiments with 10 mice per group. Each symbol represents an individual animal and the horizontal bar represents the mean. ***p < 0.001 and ****p < 0.0001 using one-way ANOVA followed by Bonferroni’s multiple comparisons test

To examine whether TGFβR1 regulation of DC homeostasis was cell intrinsic, we generated mixed bone marrow (BM) chimeric mice by reconstituting lethally irradiated (CD45.1^+^ × CD45.2^+^) WT mice with a 1:1 ratio of WT (CD45.1^+^) and either *Rag1* ^−*/*−^Cre^−^ or *Rag1* ^−*/*−^Cre^+^ (CD45.2^+^) BM cells (Fig. 4a). Under these conditions, CD103^+^CD11b^−^ DCs were produced equally efficiently from WT and transgenic BM regardless of whether *Rag1* ^−*/*−^Cre^−^ or *Rag1* ^−*/*−^Cre^+^ BM was used to generate the chimera (Fig. 4b). In contrast, whereas CD103^+^CD11b^+^ DCs derived equally from the different BM sources in WT:*Rag1* ^−*/*−^Cre^−^ chimeras, the vast majority derived from WT BM in WT:*Rag1* ^−*/*−^Cre^+^ chimeras, consistent with the reduced numbers of these cells in intact *Rag1* ^−*/*−^Cre^+^ mice (Fig. 4b). Similarly, more of the CD103^−^CD11b^+^ DC subset was derived from *Rag1* ^−*/*−^Cre^+^ BM compared with WT or *Rag1* ^−*/*−^Cre^−^ BM (Fig. 4c).

The presence of intact lymphoid organs in the BM chimeric mice allowed us to examine how DC populations in intestinal lymph nodes were affected by the absence of TGFβR1 signalling. As in the mucosa, *Rag1* ^−*/*−^Cre^+^ BM showed a selective defect in the ability to reconstitute CD103^+^CD11b^+^ DCs among the migratory (CD11c^+^MHCII^hi^) population of mesenteric lymph node (MLN) DCs in WT:*Rag1* ^−*/*−^ Cre^+^ chimeric mice compared with recipients of *Rag1* ^−*/*−^Cre^−^ BM (Fig. 4d). Consistently, there was a concomitant overrepresentation of the CD103^−^CD11b^+^ subset of migratory MLN DCs in the recipients of *Rag1* ^−*/*−^Cre^+^ BM, whereas CD103^+^CD11b^−^ MLN DCs were derived equally from *Rag1* ^−*/*−^ Cre^−^ or *Rag1* ^−*/*−^ Cre^+^ BM.

Thus, the defect in intestinal CD103^+^CD11b^+^ DCs in *Rag1* ^−*/*−^ Cre^*+*^ mice is due to cell intrinsic effects of TGFβR1 deficiency.

### TGFβR1 controls a developmental programme in CD11b^+^ DCs

**Fig. 5:**
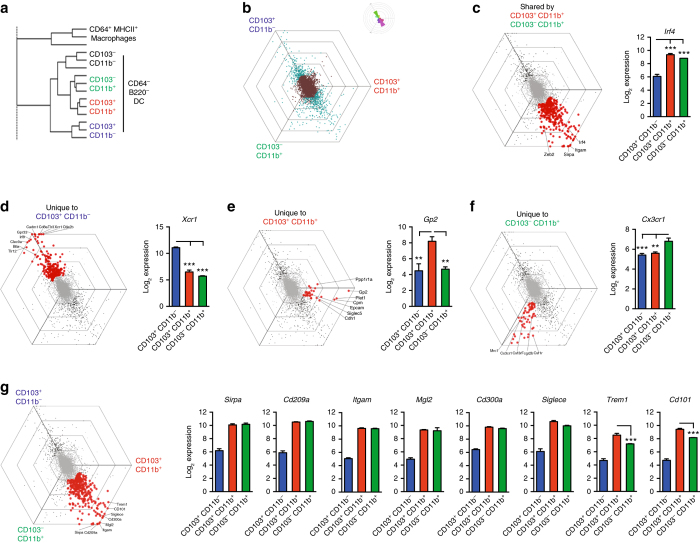
Transcriptional profiling of SILP DC reveals subset-specific markers. **a** Hierarchical clustering of DC subsets and CD64^+^MHCII^+^ macrophages from the SILP of WT mice based on microarray profiles. **b** Hexagonal ‘Triwise’ plot displaying all arrayed genes with differentially expressed genes (adj. *p*-value < 0.01, logFC > 1 or < 1) depicted in *cyan* and non-differentially expressed genes shown in *brown*. Each grid line represents a log2 fold change. Rose plots show the percentage of genes falling in each vectorial direction. **c** Triwise plot highlighting genes differentially expressed (adj. *p*-value < 0.01, logFC > 1 or < 1) by CD103^+^CD11b^*+*^ DC and CD103^−^CD11b^*+*^ DC and not CD103^+^CD11b^−^ DC (*left*) and bar chart of *Irf4* expression by DC subsets (*right*). Bars represent the mean + SEM Log_2_ expression values of three biological replicates. **d**–**f** Triwise plots highlighting genes differentially expressed (adj. *p*-value 0.01, LogFC > 1 or < 1) by CD103^+^CD11b^−^ DC **d**, CD103^+^CD11b^*+*^ DC **e** and CD103^−^CD11b^*+*^ DC **f** (*left panels*) and mean + SEM Log_2_ expression values of *Xcr1*, *Gp2* and *Cx3cr1* by DC subsets (*right panels*). **g** Triwise plots depicting the genes that are most differentially expressed by CD103^+^CD11b^*+*^ and CD103^−^CD11b^*+*^ DC subsets compared with CD103^+^CD11b^−^ DC (*left*); mean + SEM Log_2_ expression values of these genes (*right*)

As TGFβ is known to control the expression of CD103 on mucosal T cells^18, 30^, it was possible that the apparent reduction in the CD103^+^CD11b^+^ DC compartment could reflect an isolated failure to express CD103, rather than a more general effect of TGFβR1 deficiency on intestinal DC homeostasis. To distinguish between these ideas, we sought surrogate markers that were not affected by TGFβR1 deficiency and that might allow us to identify cells within the putative CD103^+^CD11b^+^ DC lineage without using CD103 itself. As a first step in this process, we used microarray analysis to compare the transcriptomes of all four of the CD103/CD11b-defined DC subsets from WT small intestine, as this information was not available from existing databases. Hierarchical clustering analysis demonstrated that the DC subsets segregated clearly from each other and from CD64^+^ mϕ (Fig. 5a). As before, we excluded the small CD103^−^CD11b^−^ population from this analysis and to visualise the differences between the remaining three DC populations, we plotted each gene in a graph comprising one axis per DC subset placed at a 120° angle to each other, creating a hexagonal ‘Triwise’ plot (Fig. 5b). In these hexagons, the distance of a point from the centre represents the magnitude of upregulation and genes that are upregulated in a particular subset are positioned close to the appropriate axis, whereas those that are shared by two subsets are found between the axes^31, 32^.

This analysis revealed that the most differentially expressed genes segregated into two main groups, one of which was associated selectively with CD103^+^CD11b^−^ DCs, whereas the other contained genes that were shared by CD103^−^CD11b^+^ and CD103^+^CD11b^+^ DCs (Figs. 5b, c). CD103^+^CD11b^−^ DCs expressed a number of genes that characterise cDC1 cells in other tissues, including *Xcr1*, *Irf8*, *Cd8a*, *Clec9a*, *Cadm1* and *Btla* (Fig. 5d and Supplementary Table 1)^9, 11, 33, 34^. The CD11b-expressing subsets of intestinal DC segregated relatively closely together in the hexagonal analysis and shared several genes typical of the conventional DC subset 2 (cDC2) lineage, including the transcription factors *Zeb2* ^32^ and *Irf4* ^6, 15, 35^, as well as *Sirpa* ^12^ (Fig. 5b and Supplementary Table 2). Nevertheless, a number of genes were also differentially expressed by the CD103^−^CD11b^+^ and CD103^+^CD11b^+^ subsets, indicating that CD103 is not the only marker that distinguishes these populations (Figs. 5e, f). Using the hexagonal analysis approach, 61 genes were found to be expressed at significantly higher levels by CD103^−^CD11b^+^ DCs, including *Cx3cr1*, *Csf1r* and *S100a4* (Fig. 5f and Supplementary Table 3). Conversely, 31 genes were expressed at significantly higher levels by the CD103^+^CD11b^+^ DC subset, including *Gp2*, *Cdh1* (encoding E-cadherin), *Siglecv* (encoding SiglecF) and *Epcam* (Fig. 5e and Supplementary Table 4). We attempted to exploit these markers for identifying the CD103^+^CD11b^+^ lineage by flow cytometry, but could not detect surface expression of E-cadherin or GP2 reliably. In addition, none of commercial antibodies against SiglecF or EpCAM permitted adequate discrimination of CD103^+^CD11b^+^ DCs from CD103^−^CD11b^+^ DCs, limiting their usefulness (Supplementary Fig. 4).

**Fig. 6:**
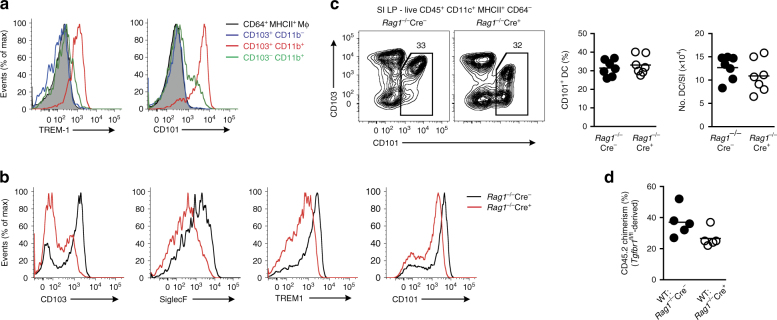
CD101 and TREM1 are surrogate markers for intestinal CD103^+^ CD11b^+^ DC. **a** Representative expression of TREM1 and CD101 by DC subsets and CD64^+^MHCII^+^ macrophages from SILP of *Rag1* ^−*/*−^Cre^−^ mice. **b** Representative expression of CD103, SiglecF, TREM1 and CD101 by total CD11b^+^ DC from the SILP of *Rag1* ^−*/*−^Cre^−^ or *Rag1* ^−*/*−^Cre^*+*^ mice. **c** Representative expression of CD103 and CD101 by total CD11c^+^MHCII^+^CD64^−^ DC (*left*) and the frequency and absolute numbers of CD101^+^ DC in the SILP of *Rag1* ^−*/*−^Cre^−^ or *Rag1* ^−*/*−^Cre^*+*^ mice. Data are pooled from two independent experiments with seven mice per group. **d** Proportion of CD45.2^+^ *Tgfbr1* ^fl/fl^-derived cells among CD101^+^ DC from WT:*Rag1* ^−*/*−^Cre^−^ or WT: *Rag1* ^−*/*−^Cre^*+*^ mixed BM chimeras 8–12 weeks post reconstitution. Data are from one of two independent experiments with five mice per group

To overcome this issue, we extended our analysis of the microarray data to include genes that, although shared by CD103^+^ and CD103^−^ CD11b-expressing DCs, were the most differentially expressed compared with the CD103^+^CD11b^−^ subset (and therefore fall on the outermost ring of the hexagonal plot in the region between the CD103^+^CD11b^+^ and CD103^−^CD11b^+^ axes). This generated an additional 34 genes (Fig. 5g and Supplementary Table 6), of which 18 encoded cell surface markers (Supplementary Table 5). Commercial antibodies were available to analyse eight of these by flow cytometry *(Mgl2*, *Siglece*, *Sirpa*, *Itgam*, *Cd300a*, *Cd209a*, *Trem1* and *Cd101)*, but *Cd101* and *Trem1* showed clear differential expression between CD103^+^ and CD103^−^ CD11b-expressing DCs at the RNA level, suggesting that they might be useful as surrogate markers (Fig. 5g). Flow cytometry showed that surface expression of TREM1 was restricted to the CD103^+^CD11b^+^ subset of SILP DCs and was present on most of these cells, as was CD101 (Fig. 6a and Supplementary Fig. 5). However, CD101 was also expressed by a fraction of CD103^−^CD11b^+^ DCs, but not by CD103^+^CD11b^−^ DCs. Similar to CD103, the expression of TREM1 by CD11b^+^ SILP DCs was TGFβR dependent, as it was markedly reduced in *Rag1* ^−*/*−^Cre^+^ mice compared with *Rag1* ^−*/*−^Cre^−^ mice (Fig. 6b). Siglec F expression by these DCs followed a similar pattern (Fig. 6b). In contrast, CD101 expression was relatively unaffected by TGFβR1 deficiency (Fig. 6b), with SILP DCs from *Rag1* ^−*/*−^Cre^+^ mice and Cre^−^ littermates containing equivalent proportions and absolute numbers of total CD101^+^ DCs (Fig. 6c). Total CD101^+^ DCs were also derived equally from *Rag1* ^−*/*−^Cre^−^ and *Rag1* ^−*/*−^Cre^+^ marrow in mixed BM chimeric mice (Fig. 6d).

Thus, the definitive phenotypic signature of intestinal CD103^+^CD11b^+^ DC requires the TGFβR1.

### Functional effects of TGFβR1 deficiency in intestinal DCs

**Fig. 7:**
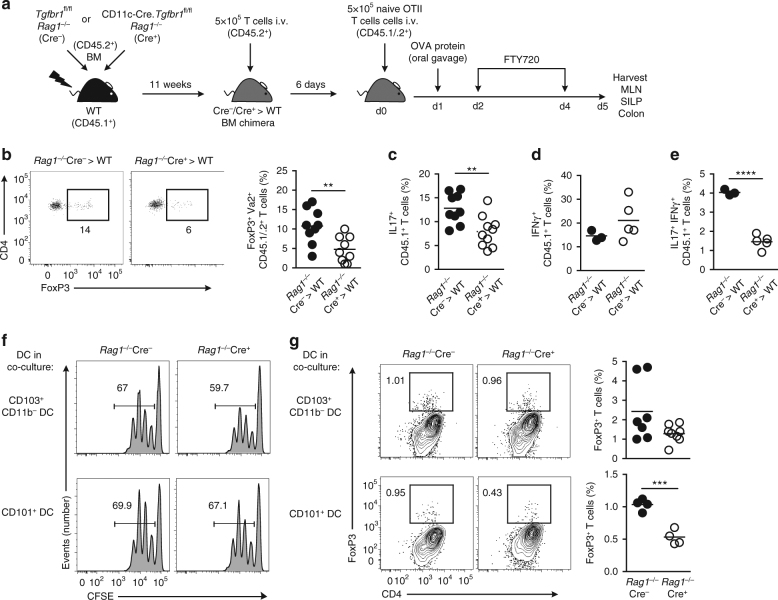
TGFβR1 signalling is required for induction of regulatory T cells by DC in vivo and in vitro. **a** Experimental scheme for induction of Tregs in vivo. **b** Representative expression of FoxP3^+^ by adoptively transferred OTII T cells (Vα2^+^CD45.1^+^CD45.2^+^) from MLN of *Rag1* ^−*/*−^Cre^−^ → WT or *Rag1* ^−*/*−^Cre^+^ → WT BM chimeric mice 4 days after oral administration of 50 mg OVA (*left panels*). Scatterplot shows the frequency of FoxP3^+^ T cells among all Vα2^+^CD45.1^+^CD45.2^+^ T cells (*right panel*). Each symbol represents an individual animal and the *horizontal bar* represents the mean. Data are pooled from two independent experiments with nine mice per group. **c**–**e** Frequency of IL17A^+^ **c**, IFNγ^+^ **d** and IL17A^+^IFNγ^+^ **e** T cells among CD45.1^+^ (residual host) T cells from the SILP of *Rag1* ^−*/*−^Cre^−^ → WT or *Rag1* ^−*/*−^Cre^+^ → WT BM chimeric mice. Data are pooled from two independent experiments with nine mice per group **c** or from one experiment with three (*Rag1* ^−*/*−^Cre^−^ → WT) or 5 (*Rag1* ^−*/*−^Cre^*+*^ → WT) mice per group **d**, **e**. **p < 0.01 and ****p < 0.0001 determined by Student’s *t*-test. CFSE profile **f** and expression of FoxP3 **g** of CD4^+^ OTII T cells after 3.5 days of co-culture with FACS-purified CD103^+^CD11b^−^ DC or CD101^+^ DC from SILP of *Rag1* ^−*/*−^Cre^−^ or *Rag1* ^−*/*−^Cre^*+*^ mice in the presence of 0.5 μg ml^−1^ OVA 323–339 peptide. Data are pooled from at least four individual experiments, with each symbol representing a biological replicate and the horizontal bar representing the mean. ***p < 0.001 determined by Student’s *t*-test

Depending on the experimental system, CD103^+^CD11b^+^ DCs have been implicated in the generation of both Th17 cells and FoxP3^+^ Treg^6, 15, 35–38^. Thus, we assessed whether TGFβR1-mediated deficiency in this DC lineage resulted in functional defects in T cells. To examine Treg priming in vivo, we transferred naïve ovalbumin (OVA)-specific OTII CD4^+^ T cells into *Rag1* ^−*/*−^Cre^−^ → WT and *Rag1* ^−*/*−^ Cre^*+*^ → WT BM chimeric mice and assessed the induction of FoxP3-expressing cells in MLN 4 days after oral administration of OVA (Fig. 7a). Notably, there were significantly fewer OVA-specific FoxP3^+^ Treg in the MLNs of *Rag1* ^−*/*−^Cre^+^ → WT chimeras compared with *Rag1* ^−*/*−^Cre^−^ → WT chimeras (Fig. 7b). As both CD103^+^CD11b^+^ and CD103^+^CD11b^−^ DCs have been shown to be able to induce Treg differentiation, we next assessed the relative capabilities of these subsets in vitro. Purified small intestinal CD101^+^ DCs or CD103^+^CD11b^−^ DCs from *Rag1* ^−*/*−^Cre^−^ or *Rag1* ^−*/*−^Cre^+^ mice were loaded with OVA peptide and co-cultured with CFSE-labelled naive OTII CD4^+^ T cells. Both DC subsets, irrespective of the presence or absence of TGFβR1, induced T-cell proliferation, as measured by CFSE dilution (Fig. 7f). However, whereas CD103^+^CD11b^−^ DCs from *Rag1* ^−*/*−^Cre^−^ and *Rag1* ^−*/*−^Cre^+^ mice induced equivalent levels of antigen-specific FoxP3^+^ Treg, TGFβR1-deficient CD101^+^ DCs induced 50% fewer FoxP3^+^ Treg when compared with CD101^+^ DCs from *Rag1* ^−*/*−^Cre^−^ littermates (Fig. 7g).

Feeding OVA alone does not induce high frequencies of effector T cells in vivo^35^. However, residual endogenous T cells were present in our chimeric mice and analysis of these cells showed a significant reduction in the numbers of IL-17A-producing CD4^+^ T cells in the small intestinal mucosa of *Rag1* ^−*/*−^Cre^+^ → WT chimeras compared with *Rag1* ^−*/*−^Cre^−^ → WT chimeras (Fig. 7c). Similarly, IL-17A^+^IFNγ^+^ CD4^+^ T cells, which have been shown to derive from IL-17A^+^IFNγ^−^ CD4^+^ T cells^39^, were also reduced in the *Rag1* ^−*/*−^Cre^+^ → WT chimeric intestine, whereas IL-17A^−^IFNγ^+^ T cells were present in equal proportions in the two groups (Figs. 7d, e). Collectively, these data demonstrate that TGFβR1 signalling is essential for complete maturation of fully functional CD103^+^CD11b^+^ DCs in the intestine.

## Discussion

CD103^+^CD11b^+^ DCs are the predominant population of DCs in the small intestine and are virtually unique to the intestinal mucosa and its draining lymphoid tissues^40^. Although it is known that CD103^+^CD11b^+^ DCs derive from conventional DC progenitors that mature locally after their arrival in the intestine^6, 41^, the environmental factors responsible for the development of these DCs remain poorly understood. We show here that CD103^+^CD11b^+^ DCs are transcriptionally closely related to their CD103^−^CD11b^+^ counterparts. However, unlike CD103^−^CD11b^+^ DCs, cell intrinsic TGFβR1 signalling is indispensable for the full phenotypic differentiation of CD103^+^CD11b^+^ DC in the SILP. Furthermore, using whole-genome analysis and flow cytometric validation, we demonstrate that differentiation to the CD103^+^CD11b^+^ phenotype represents more than simple acquisition of CD103 expression and involves significant changes in gene expression. Finally, we demonstrate that aborted DC differentiation in the absence of TGFβR1 signalling renders DCs less able to prime Treg and maintain Th17 in vivo, demonstrating that TGFβR1 signalling in DCs helps maintain immunological homeostasis in the intestine.

We first attempted to address the role of the TGFβ–TGFβR axis in intestinal DC homeostasis by generating CD11c*-*Cre*.Tgfbr1* ^fl/fl^ mice, but these developed a lethal systemic inflammatory disease, with all animals dying within 20 weeks. Similar findings were made in mice with CD11c-driven deletion of *Tgfbr2* ^26^, but in contrast to that work the wasting disease in our mice was predominantly T-cell driven. This was shown by the fact that T cells in CD11c*-*Cre.*Rosa26*-LSL-YFP mice displayed Cre recombinase activity and there was deletion of the *Tgfbr1* in T cells from Cre^+^ CD11c*-*Cre*.Tgfbr1* ^fl/fl^ mice. In support of this, the wasting disease in Cre^+^ mice was prevented by maintaining mice on a *Rag1* ^−*/*−^ background and T cells purified from Cre^+^ mice were able to induce disease in WT mice where they underwent uncontrolled expansion. Similar autoimmune disease was found in mice with CD4-driven deletion of *Tgfbr2* ^28, 42^. Together, our findings are consistent with the Cre activity found in T cells in the original description of CD11c*-*Cre mice^25^ and they underline the need for extreme caution when interpreting studies employing CD11c-dependent strategies to dissect ‘DC’ function in vivo.

TGFβR1 also controlled the behaviour of CD11c^+^ cells other than T cells, as intestinal inflammation developed in *Rag1* ^−*/*−^Cre^+^ recipients of WT T cells and this was associated with a selective defect in CD103^+^CD11b^+^ DCs in the intestine of *Rag1* ^−*/*−^ CD11c*-*Cre*.Tgfbr1* ^fl/fl^ mice. Other mononuclear phagocytes whose homeostasis is controlled by the TGFβ–TGFβR1 axis include microglia in the brain and Langerhans cells in the epidermis^20, 22, 43, 44^. Interestingly, TGFβ regulates expression of E-cadherin by Langerhans cells^23, 45^ and *Cdh1* was one of the genes we found to be selectively upregulated in CD103^+^CD11b^+^ DCs. However, our findings contrast with those from the skin, where TGFβR signalling is required for both Langerhans cell development and for preventing their spontaneous ‘maturation’ and enhanced migration to draining lymph nodes^23, 43^. An analogous process cannot explain the lack of CD103^+^CD11b^+^ DCs in the intestine, as this population was not overrepresented in the draining MLN of *Rag1* ^−*/*−^Cre^+^ → WT BM chimeras, as would be the case if they had acquired enhanced migratory potential. Furthermore, the total DC pool in the intestine was equivalent in *Rag1* ^−*/*−^Cre^−^ and *Rag1* ^−*/*−^Cre^+^ mice. Thus, we favour the idea that there is aborted differentiation of CD103^+^CD11b^+^ DCs in the absence of TGFβR1 signalling. This was supported by our use of CD101 expression to interrogate the CD11b^+^ lineage of DCs, where it appeared to be a marker of those cells that are likely to differentiate locally into CD103^+^CD11b^+^ DCs. This was first suggested by our transcriptional analysis, which showed that the CD103^+^CD11b^+^ and CD103^−^CD11b^+^ DCs clustered closely together and identified CD101 as one of the genes that was selectively upregulated on the CD103^+^CD11b^+^ subset. Phenotypic analysis confirmed the expression of CD101 protein on virtually all WT CD103^+^CD11b^+^ DCs, but showed that a small subset of CD103^−^CD11b^+^ DCs also expressed CD101. The fact that these might be the immediate precursors of CD103^+^CD11b^+^ DCs would be consistent with the reciprocal increase we found in the CD103^−^CD11b^+^ CD101^+^ population of DCs in CD11c*-*Cre*.Tgfbr1* ^fl/fl^ mice. Although TGFβ has been shown previously to induce the expression of CD103 on T cells^18, 30^, the block in DC development that occurs in the absence of TGFβR1 signalling in vivo did not simply reflect a failure to upregulate CD103, as CD101^+^CD11b^+^ DCs in CD11c*-*Cre*.Tgfbr1* ^fl/fl^ mice also had reduced expression of SiglecF and TREM1, both of which we found to be specific to this subset at the transcriptional and protein level. On the basis of these findings, we propose that TGFβ is important for the maturation of CD103^+^CD11b^+^ DCs from an earlier CD103^−^CD11b^+^ stage. However, definitive proof of this idea is lacking and it will be important to establish at exactly what stage TGFβ acts. As we found that all the subsets of mature DCs express the TGFβR1, the selective conditioning effects of TGFβ may occur as DC precursors mature in the mucosa^6^. Alternatively, there may be an anatomically defined niche in which CD11b^+^ DCs encounter cells producing sufficient amounts of TGFβ to trigger signalling via the TGFβR. Given the ubiquitous nature of this cytokine, such cells could be of myeloid, mesenchymal or epithelial origin in our *Rag1* ^−*/*−^ mice which lack T and B lymphocytes. Resolving these questions will require direct tracking of individual cells within the CD11b lineage, as they differentiate in the mucosa in the presence and absence of TGFβR signalling and these issues are the subject of ongoing work.

Others have reported that CD101 is expressed by some CD11b^+^ DCs in mouse intestine and that this is retinoic acid receptor dependent^16, 46^. Although it may also inhibit DC function^47^ and enhance the production of IL-10 by CD11c^+^ myeloid cells^48^, its role in DC development or function in the intestine remains to be defined. CD101 expression has also been linked to the expansion and function of Tregs, including those which can prevent experimental colitis^48, 49^, as well as a susceptibility gene in the development of type 1 diabetes in non-obese diabetic mice^50^. As CD103^+^CD11b^+^ DCs in the human intestine also express CD101^46^ and we found varying numbers of CD11b^+^ DCs to expressed CD101 in other organs, this molecule may provide a useful means for interrogating the tissue specific differentiation of the cDC2 lineage across species. Similarly, the role of TREM1 and SiglecF on CD103^+^CD11b^+^ DCs remains unclear, although our findings that these proteins are expressed preferentially by this subset extend other work suggesting this association at the transcriptional level^16^. Recently, TREM1 has been associated with inflammatory monocytes, where it modulates the function of TLR4^51^. However, it has also been reported to be present on Langerhans cells and to be upregulated on Langerhans cells and BM DCs by hypoxia^52^, suggesting it may have novel roles on MPs associated with barrier surfaces, especially under conditions of stress.

The selective loss of intestinal CD103^+^CD11b^+^ DCs in CD11c*-*Cre*.Tgfbr1* ^fl/fl^ mice is similar to that reported in mice deficient in CSF2, CSF2R^13^, IRF4^15, 35^, SIRPα^17^, Notch signalling^14, 53^ and retinoic acid production^16, 54^. However, these studies did not apply additional discriminatory markers such as CD101, TREM1 or SiglecF, and some did not distinguish bona fide CD103^−^CD11b^+^ DCs from CD11b^+^ CD64/F4/80^+^ macrophages. As macrophages are much more numerous in LP^6, 55^, their inclusion could have masked any reciprocal accumulation of CD103^−^CD11b^+^ DCs. Therefore, it is unclear whether the TGFβ–TGFβR axis interacts or synergises with any of these other pathways or whteher the absence of each of the molecules interrupts differentiation of the CD11b^+^ DC lineage at the same stage. Previous studies have implicated the transcription factor RUNX3 in mediating TGFβ dependent effects in DCs^56^, but whether RUNX3 drives the TGFβ-dependent effects reported here remains unclear.

The exact role of intestinal CD103^+^CD11b^+^ DCs has been somewhat unclear, due to apparently contradictory findings from in vitro and in vivo experiments. Whereas this population produces substantial amounts of RA and induces the generation of FoxP3^+^ Tregs in vitro^55, 57^, a universal finding from the previous studies of mice lacking CD103^+^CD11b^+^ DCs was reduced numbers of Th17 cells in vivo, with normal numbers of FoxP3^+^ Tregs^6, 15, 35, 37^ The relative distribution of CD103^+^CD11b^+^ DCs along the length of the intestine also correlates with that of Th17 cells and inversely with that of FoxP3^+^ Tregs^55^. Therefore, it was notable that we found here that the reduction in CD103^+^CD11b^+^ DCs in CD11c*-*Cre*.Tgfbr1* ^fl/fl^ mice was not only associated with a lower proportion of endogenous Th17 cells in the intestinal mucosa, but there also was defective generation of antigen specific FoxP3^+^ Tregs, both in vitro and in vivo. The mechanism underlying these effects remains to be elucidated, but TGFβ is important in the generation of both Treg and Th17 cells^36, 58^. Therefore, one possibility is that TGFβR signalling in CD103^+^CD11b^+^ DCs normally drives TGFβ production via a positive feedback circuit, as has been shown for other DCs^59^. Although our findings show that the defect in CD103^+^CD11b^+^ DCs was associated with reduced numbers of Treg contrasts with the earlier studies, it is important to note that we focused directly on Tregs that were induced in response to their cognate antigen. In contrast, previous work either assessed total Treg numbers, or used surrogate markers to define ‘natural’ and ‘inducible’ Tregs. Alternatively, CD103^+^CD11b^−^ DCs might compensate for the loss of CD103^+^CD11b^+^ DCs in the induction of oral tolerance and Treg,^37, 60^ and, importantly, we found no defect in the ability of CD103^+^CD11b^−^ DCs from Cre^+^ mice to induce Treg in vitro. However, as we did not assess the suppressive functions of these Tregs directly, we cannot rule out the possibility that the FoxP3^+^ T cells we found to be generated by CD103^+^CD11b^−^ DCs from Cre^+^ mice have altered activity despite being present in normal numbers. Furthermore, it is unclear whether the defect in Treg we found contributes to the autoimmunity found in immunocompetent CD11c*-*Cre*.Tgfbr1* ^fl/fl^ mice. Discrepancies between different labs on how defects in CD103^+^CD11b^+^ DCs might impact on T-cell subsets could also reflect the microbiota present in the various strains or animal facilities. For instance, the ability of CD103^+^CD11b^+^ DC to produce IL-23 and drive the differentiation of Th17 cells in vitro can be promoted by TLR5 ligation^38, 61^. Alternatively, the different strategies used to target CD103^+^CD11b^+^ DCs may have distinct consequences for T-cell differentiation and, in particular, our findings that CD11c-mediated Cre activation can have effects on cells other than this specific subset of DC must be taken into account. This could help explain, for example, why contrasting results have been reported on the generation of protective Th17 responses to *Citrobacter rodentium* infection when CD103^+^CD11b^+^ DCs are lacking in human Langerin-DTA transgenic mice and CD11c-Cre.*Notch2* ^fl/fl^ mice^37, 53^. Further work is required to elucidate directly how this subset of DC is influenced by different environmental factors and targeting strategies.

## Methods

### Mice

CD11c*-*Cre.*Tgfbr1* ^fl/fl^ mice on a C57BL/6 background were generated as described previously^23^ and crossed to *Rag1* ^−*/*−^ mice (Jackson Laboratories) to generate *Rag1* ^−*/*−^ CD11c*-*Cre.*Tgfbr1* ^fl/fl^ mice. Cre^−^ littermates were used as experimental controls in all the experiments presented. WT C57BL/6 (CD45.2^+^) mice were obtained from Harlan Olac (Bicester, UK), whereas C57BL/6.SJL (CD45.1^+^) mice, C57BL/6.SJL × C57Bl/6 (CD45.1^+^ × CD45.2^+^) and OTII OVA-specific TcR transgenic mice on the CD45.1^+^ background were bred in house. All mice were used at 6–12 weeks of age, and male and female mice were used throughout the study. Transgenic and control mice were sex-matched within experiments. All mice were bred and maintained in specified pathogen-free conditions at the Central Research Facility at the University of Glasgow under a UK Home Office Project Licence and approved by the University of Glasgow Local Ethical Review Panel, or at the University Medical Centre, Rotterdam, and approved by the Animal Experiments Committee DEC–Consult of the Erasmus University Medical Center Rotterdam. No randomization or blinding was performed.

### Generation of bone marrow chimeric mice

To generate mixed chimeras, 8–10-week-old CD45.1^+^ WT mice were lethally irradiated with two doses of 5 Gy 1 h apart before being reconstituted immediately with 5 × 10^6^ BM cells from CD45.2^+^ *Rag1* ^−*/*−^CD11c*-*Cre*.Tgfbr1* ^fl/fl^ or *Rag1* ^−*/*−^ *Tgfbr1* ^fl/fl^ mice together with CD45.1^+^ × CD45.2^+^ WT BM at a ratio of 1:1. In some experiments, CD45.1^+^ × CD45.2^+^ mice were used as the recipient mice and CD45.1^+^ WT mice used as the WT donor. To generate full chimeras, CD45.1^+^ WT mice were given BM cells from CD45.2^+^ *Rag1* ^−*/*−^CD11c*-*Cre*.Tgfbr1* ^fl/fl^ or *Rag1* ^−*/*−^ *Tgfbr1* ^fl/fl^ mice. Chimerism was assessed at least 8 weeks after reconstitution.

### Generation of Treg cells in vivo

Eight to 10 weeks after reconstitution, *Rag1* ^−*/*−^ CD11c*-*Cre*.Tgfbr1* ^fl/fl^ and *Rag1* ^−*/*−^ *Tgfbr1* ^fl/fl^ mice → CD45.1^+^ WT chimeric received 5 × 10^5^ purified CD4^+^ T cells from the spleen and lymph nodes of CD45.2^+^ WT mice intravenously. Six days later mice were adoptively transferred with 5 × 10^5^ fluorescence-activated cell sorting (FACS) sorted naive CD4^+^CD62L^+^CD45.1^+^/CD45.2^+^ OT II cells intravenously, before being fed 50 mg OVA 24 h later. FTY720 (1 mg kg^*−*1^; Cayman Chemical Company) was injected intraperitoneally 2 and 4 days after OTII T-cell transfer and mice were culled 5 days after T-cell transfer for assessment of Foxp3^+^ expression by CD4^+^Vα2^+^ transgenic T cells in mesenteric lymph nodes using intracellular FACS analysis.

### Induction of T-cell-dependent colitis

CD3^+^MHC-II^neg^ T cells were sorted from the spleens of C57BL/6 CD45.1 mice, resuspended in sterile phosphate-buffered saline (PBS) and 5 × 10^6^ T cells were injected intravenously into *Rag1* ^−*/*−^ CD11c*-*Cre.*Tgfbr1* ^fl/fl^ (*Rag1* ^−*/*−^Cre^+^) mice and *Rag1* ^−*/*−^ *Tgfbr1* ^fl/fl^ (*Rag1* ^−*/*−^Cre^−^) littermates. Body weight was monitored twice a week and the recipients were killed when 20% weight loss occurred (10–17 weeks post transfer). The severity of bowel inflammation was evaluated by scoring of histological sections (adapted from ref. ^62^).

### Preparation of single-cell suspensions

To isolate small intestinal leukocytes, small intestine were flushed with calcium/magnesium-free (CMF) Hank’s balanced salt solution (HBSS) 2% fetal calf serum (FCS) (both Gibco, Invitrogen, Paisley, UK) and PPs excised. The intestines were opened longitudinally, washed again in HBSS 2% FCS and cut into 0.5 cm segments, which were then incubated twice in HBSS with 2 mM EDTA at 37 °C with shaking for 20 min. Supernatants were discarded and intestinal tissue digested with 1 mg ml^*−*1^ collagenase VIII (Sigma-Aldrich) in complete RPMI 1640 containing 2 mM l-glutamine, 100 μg ml^*−*1^ penicillin, 100 μg ml^*−*1^ streptomycin, 1.25 μg ml^*−*1^ Fungizone and 10% FCS (all Gibco, Invitrogen) at 37 °C with shaking for 20 min. Cell suspensions were passed through 100 μm and then 40 μm filters (BD Falcon) and stained for flow cytometry. To isolate colonic leukocytes, colons were excised and soaked in PBS. After removing all fat and faeces, the colons were opened longitudinally, washed in HBSS 2% FCS and cut into 0.5 cm sections. The tissue was then shaken vigorously in 10 ml HBSS/2% FCS and the supernatant was discarded. To remove the epithelial layer, 10 ml CMF HBSS containing 2 mM EDTA was then added, the tube placed in a shaking incubator for 15 min at 37 °C, before being shaken vigorously and the supernatant discarded. Tissue segments were washed in 10 ml fresh CMF HBSS, before a second incubation in CMF HBSS/2 mM EDTA, the wash step was repeated and the remaining tissue was digested with pre-warmed ‘enzyme cocktail’ containing 1.25 mg ml^*−*1^ collagenase D (Roche), 0.85 mg ml^*–*1^ collagenase V (Sigma-Aldrich), 1 mg ml^−1^ dispase (Gibco, Invitrogen) and 30 U ml^−1^ DNase (Roche Diagnostics GmbH, Mannheim, Germany) in complete RPMI 1640 for 30–45 min in a shaking incubator at 37 °C. The resulting cell suspension was passed through a 40-μm cell strainer and washed twice in FACS buffer (2% FCS/2mm EDTA/PBS). Cells were counted and stained for flow cytometry. To isolate leukocytes from MLNs, MLNs were minced with scissors and incubated with 1 mg ml^−1^ collagenase D in CMF HBSS for 45 min in a shaking incubator at 37 °C. After digestion, cells were passed through a 100-μm filter and kept on ice until further use^63^. Lungs were removed from perfused mice, chopped finely and digested in pre-warmed ‘enzyme cocktail’ for 45 min in a shaking incubator at 37 °C before being passed through an 100 μm strainer followed by centrifugation at 400 *g* for 5 min. Spleens were chopped finely and digested in HBSS with 1 mg ml^−1^ collagenase D for 45 min in a shaking incubator at 37 °C before being passed through a 100 μm strainer followed by centrifugation at 400 g for 5 min. Cells were resuspended in FACS buffer, counted and kept on ice until staining for flow cytometry.

### T cell and DC co-cultures

A total of 12,500 CD45^+^CD11c^+^MHCII^+^CD64^−^CD101^+^CD11b^+^ DCs were FACS purified from the SILP of *Rag1* ^−*/*−^CD11c*-*Cre*.Tgfbr1* ^fl/fl^ or *Rag1* ^−*/*−^ *Tgfbr1* ^fl/fl^ mice before being pulsed with 0.5 μg ml^−1^ of OVA323-339 peptide (Genosys, Sigma) in 100 μl for 30 min at 37 °C in 5% CO_2_ in RPMI 1640 containing 2 mM l-glutamine, 100 μg ml^−1^ penicillin, 100 μg ml^−1^ streptomycin, 1.25 μg ml^−1^ Fungizone and 10% FCS; all from Gibco) in 96-well round-bottom plates. After 30 min, 1 × 10^5^ CFSE-labelled naive CD4^+^CD62L^+^ T cells FACS sorted from the lymph nodes of OTII.CD45.1^+^ TcR transgenic mice were added and cultured for 3.5 days at 37 °C in 5% CO_2_. After culture, responding OTII cells were assessed for expression of FoxP3 and CFSE dilution by flow cytometry.

### Flow cytometry

Following incubation with purified anti-CD16/CD32 for 10 min at 4 °C, 1−10 × 10^6^ cells were stained at 4 °C in the dark as described previously^63, 64^ using the antibodies listed in Supplementary Table 6 and analysed using an LSR II or FACSAriaI/III cytometer (BD Biosciences) and FlowJo software (Tree Star).

### Genomic PCR

Cre-mediated deletion of *Tgfbr1* was analysed by genomic PCR^65^. The floxed third exon of *Tgfbr1* was identified by a 250-bp band, whereas excision was demonstrated by a 350-bp band (see Supplementary Table 7).

### Quantitative RT-PCR

mRNA was extracted from cells using the GenElute mammalian total RNA Miniprep kit (Sigma-Aldrich) and complementary DNA was synthesized with SuperScript II reverse transcriptase (Invitrogen), according to the manufacturer’s protocol. TaqMan real-time quantitative PCR assays were designed using Universal ProbeLibrary (Roche) to determine transcript levels of the indicated genes using the primers listed in Supplementary Table 8. Expression levels were normalized to the control gene *Gapdh* or *Abl* as indicated. All reactions were run on a 7900HT Fast Real Time PCR machine (Applied Biosystems).

### Histology

Tissues were snap-frozen, cut into 6 µm sections and fixed in acetone/0.02% H_2_O_2_. For detection of CD3 (Dako Heverlee) and Ki67 (Novocastra), endogenous peroxidases were quenched with 3% H_2_O_2_ in methanol for 20 min. After microwaving in citrate buffer (10 mM, pH 6.0) for antigen retrieval, sections were blocked for 1 h in 10 mM Tris, 5 mM EDTA, 0.15 M NaCl, 0.05% Tween-20 and 10% normal mouse serum (NMS), before being stained overnight with primary antibodies against CD4 (GK1.5) and CD8 (Lyt2) (both from Dako) at 4 °C in PBS/0.1% BSA. After washing, biotinylated rabbit anti-rat secondary antibody (Dako) or goat anti-rabbit serum was added in PBS/0.1% BSA/2% NMS for 1 h at room temperature. Enzyme activity was revealed by using the Vectastain ABC kit (Vector Laboratories). Aminoethylcarbazole (Sigma) or 3,3′-diaminobenzidine tetrahydrochloride (Sigma-Aldrich) was used as a chromogen for horseradish peroxidase activity. The sections were counterstained with haematoxylin and mounted with glycerol-gelatin. The slides were scanned with the NanoZoomer 2.0HT scanner (Hamamatsu) and analysed with the NanoZoomer Digital Pathology programme.

### Microarray analysis

A total of 100,000 cells from each DC subset and CD45^+^Ly6C^−^MHCII^+^CD64^+^ CD11b^+^ macrophages were FACS purified from SI LP and total RNA extracted with the RNeasy Micro kit (Qiagen). Quantity, quality and absence of genomic DNA were assessed using a Bioanalyser (Agilent). For microarray analysis, 50–450 ng of total RNA was retrieved per biological sample, with RIN values always above 8.25 ng total RNA was used for each sample for synthesis of biotinylated double stranded cDNA, using the NuGEN Ovation Pico WTA System V2 kit and the NuGEN Encore Biotin Module kit, according to the manufacturer’s recommendations. Following fragmentation and end-labelling, 2 µg of cDNA were hybridized for 16 h at 45 °C on GeneChip Mouse Gene 1.0 ST arrays (Affymetrix), interrogating 28,853 genes represented by ~27 probes spread across the full length of the gene. The chips were washed, stained in the GeneChip Fluidic Station 450 (Affymetrix) and scanned with the GeneChip Scanner 3,000 7 G (Affymetrix) at a resolution of 0.7 µm. Raw data (.cel intensity files) were extracted from the scanned images using the Affymetrix GeneChip Command Console version 3.2. After quality control using R/Bioconductor, the Robust Multi-array Average procedure was used to normalize data within arrays (probeset summarization, background correction and log2-transformation) and between arrays (quantile normalization). Only probesets that mapped uniquely to one gene were used and for each gene, the probeset with the highest expression level was used.

### Cytometric bead assay

Splenocytes (1 × 10^6^) were cultured overnight in 500 µl medium and supernatants were stored at −20 °C before analysis of IL2, IL17A and IFNγ levels using a cytometric bead assay (Bender MedSystems) with a FACS Canto-II (BD Biosciences) and FCAP Array software (BD Biosciences).

### Statistics

Statistical analyses were performed with GraphPad Prism. Student’s *t*-test was used to compare two groups, while multiple groups were compared using analysis of variance or Student’s *t*-tests with appropriate corrections as detailed in the legends. A *p*-value of 0.05 was considered significant.

### Data availability

Microarray data that support the findings of this study have been deposited in National Center for Biotechnology Information Gene Expression Omnibus public database (http://www.ncbi.nlm.nih.gov/geo/) with the primary accession code GSE100393. The other data that support the findings of this study are available from the corresponding authors upon request.

## Electronic supplementary material

Supplementary information
