# Supplementary material for: TGFβR signalling controls CD103+CD11b+ dendritic cell development in the intestine
Source: Nat Commun. 2017 Sep 20;8:620. doi: 10.1038/s41467-017-00658-6 (PMC5607002; doi:10.1038/s41467-017-00658-6)
Supplement: Supplementary file 1 — Supplementary information [file 41467_2017_658_MOESM1_ESM.pdf]

Description of Supplementary Files

File Name: Supplementary Information

Description: Supplementary figures, supplementary tables

Supplementary Figure 1

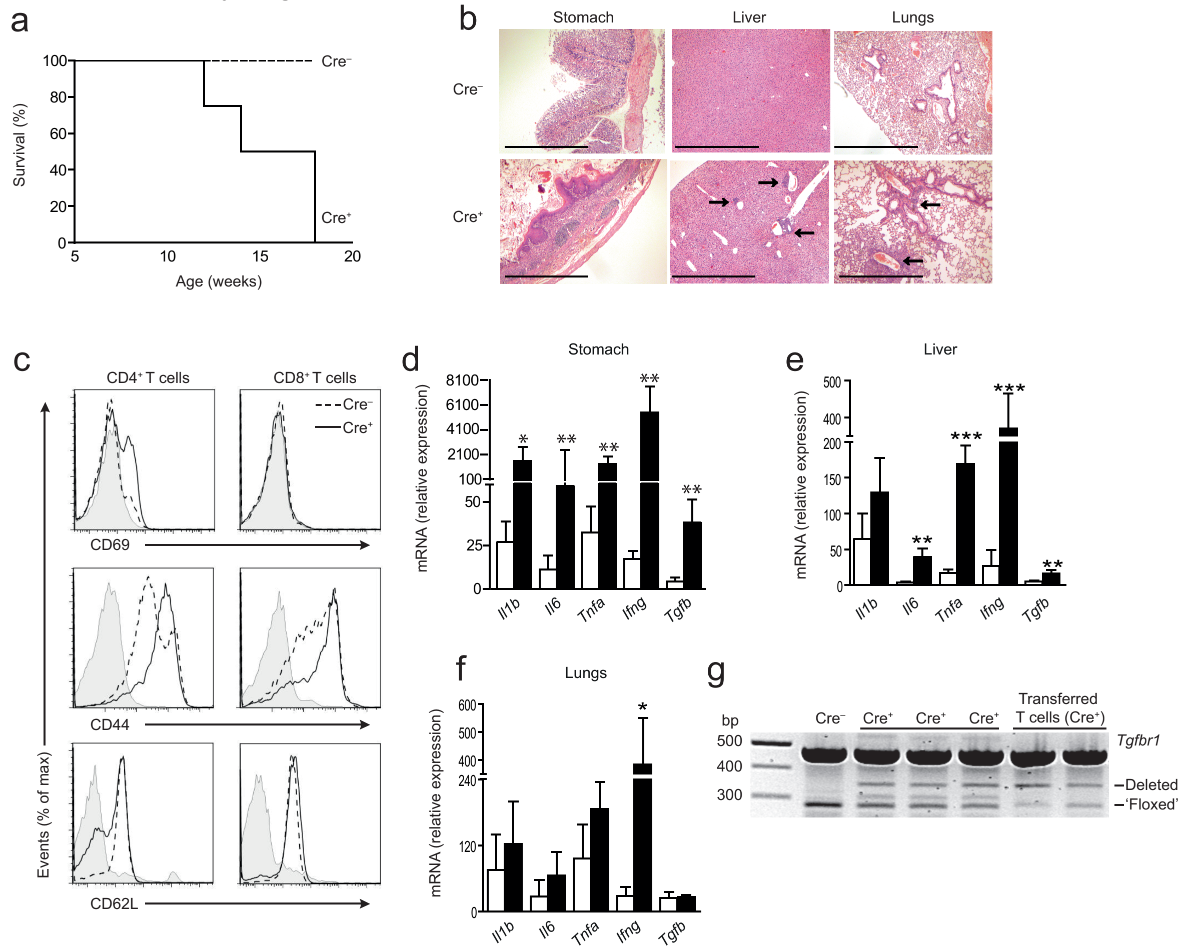

Fig. S1: CD11c-Cre.*Tgfbr1*$^{fl/fl}$ Mice Develop a Lethal Systemic Inflammatory Disease

(**a**) Survival of lymphocyte-sufficient CD11c-Cre.*Tgfbr1*$^{fl/fl}$ (Cre^{+}) or *Tgfbr1*$^{fl/fl}$ (Cre^{-}) mice with 5 (Cre^{+}) or 6 (Cre^{-}) mice/group and data are representative of 2 experiments. (**b**) H&E staining of stomach, liver and lungs from Cre^{-} (*upper panels*) or Cre^{+} (*lower panels*) mice. Images are representative of at least 4 mice/group. Scale bars 500µm. (**c**) Expression of CD69, CD44 and CD62L by splenic CD4^{+} and CD8^{+} T cells from Cre^{-} (*dashed line*) or Cre^{+} mice (*solid line*). Shaded histograms represent staining with appropriate isotype controls. (**d-f**) qRT-PCR analysis of IL1β, IL6, TNFα, IFNγ and TGFβ mRNA in stomach (**d**), liver (**e**) and lungs (**f**) of Cre^{-} or Cre^{+} mice. (**g**) Genomic PCR for *Tgfbr1* expression by splenic CD3^{+} T cells from intact lymphocyte-sufficient Cre^{-} or Cre^{+} mice, or by donor CD3^{+} T cells purified from the recipients of Cre^{+} splenic T cells ('Transferred T cells'). Flow cytometric analysis from one of two independent experiments with n=4-8/group.

Supplementary Figure 2

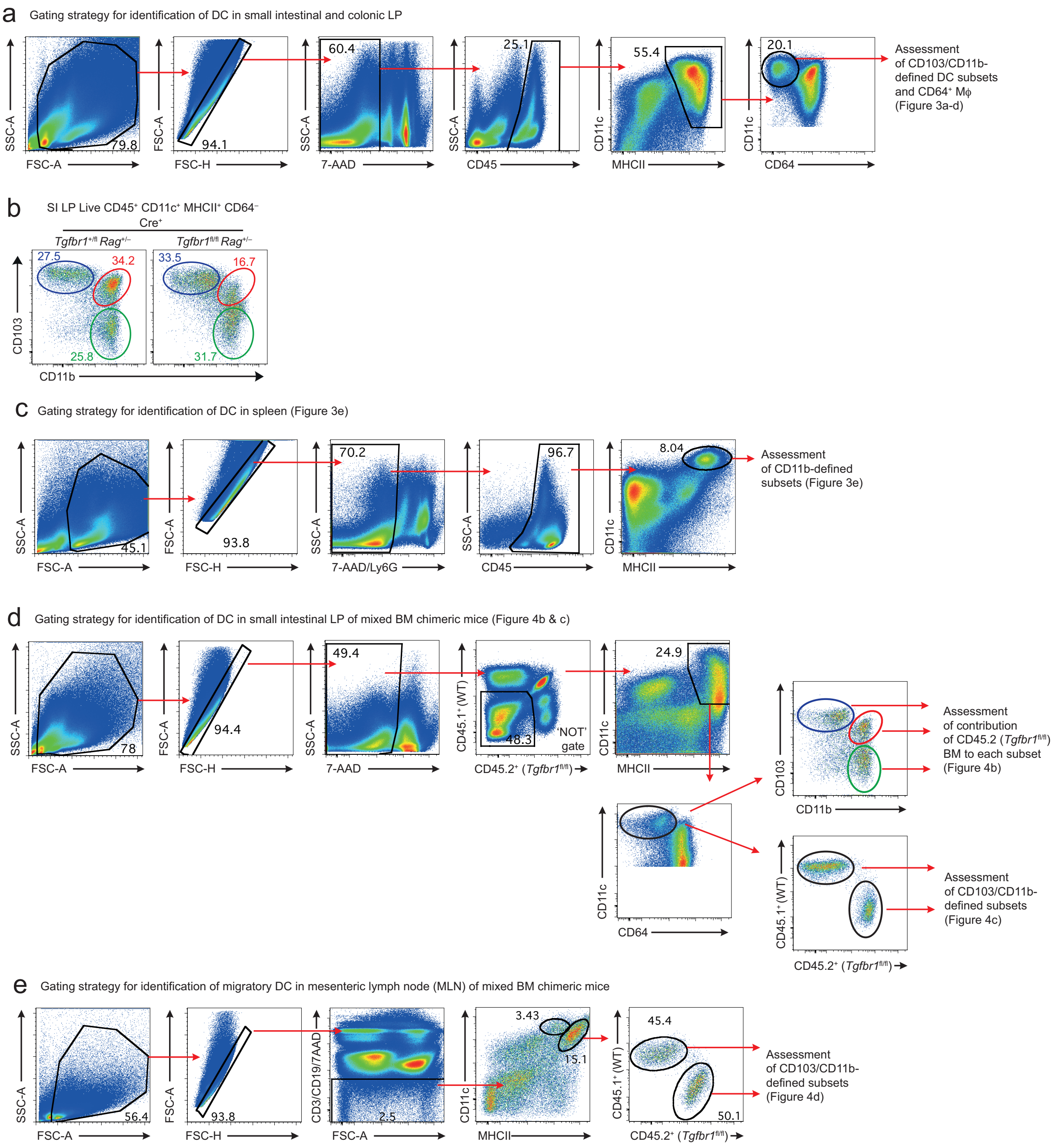

Fig. S2: Gating strategies used throughout study

(**a**) Gating strategy to identify DC and macrophages in the small intestine and colonic lamina propria in Figure 3 and Figure 6. (**b**) Representative expression of CD103 and CD11b by DC subsets from the small intestine of CD11c-Cre.*Tgfbr1*$^{+/fl}$ *Rag1*$^{+/-}$ or CD11c-Cre.*Tgfbr1*$^{fl/fl}$ *Rag1*$^{+/-}$ mice. Plots are from one experiment of two independent experiments performed with 3 mice per group. (**c**) Gating strategy to identify DC in the spleen in Figure 3. (**d**) Gating strategy to identify DC subsets in the small intestinal lamina propria of mixed BM chimeric mice in Figure 4. (**e**) Gating strategy to identify DC subsets in the mesenteric lymph node (MLN) of mixed BM chimeric mice in Figure 4.

Supplementary Figure 3

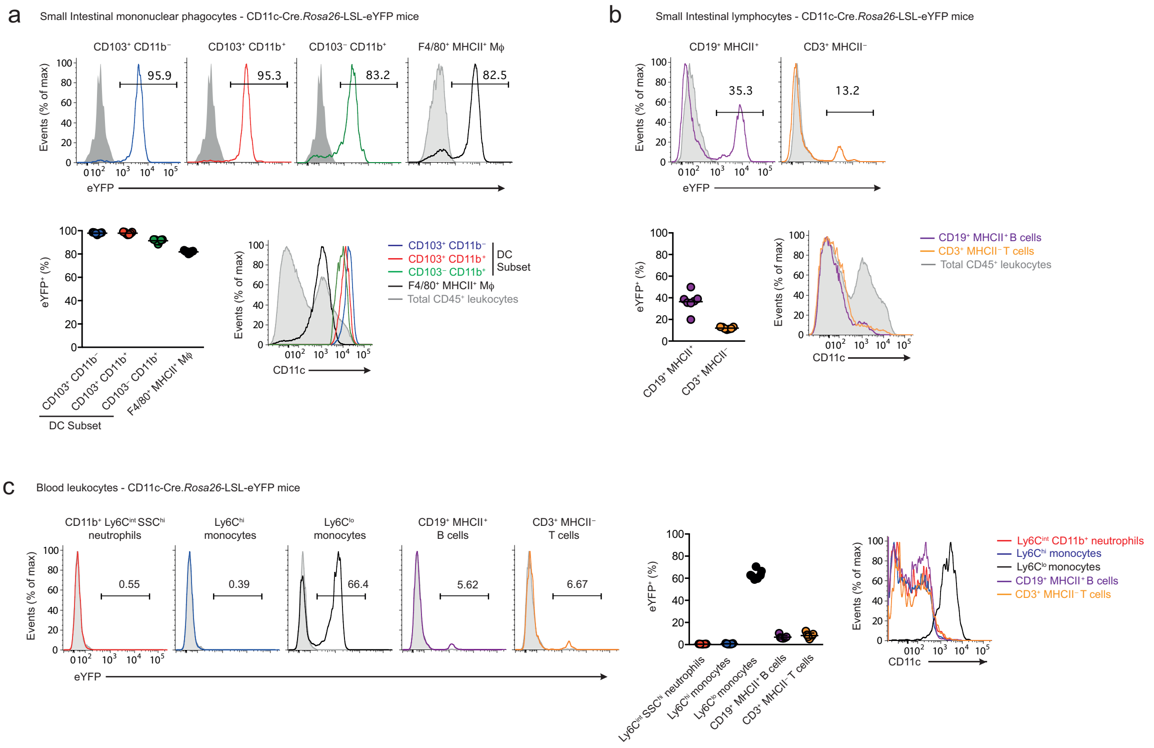

Fig. S3: Efficiency of CD11c-Driven Cre Recombinase Activity

(**a**) Representative expression of eYFP by DC subsets and F4/80^{+}MHCII^{+} macrophages from the small intestine of CD11c-Cre.*Rosa26*-LSL-eYFP mice or CD11c-Cre mice (shaded histograms). Scatter plots show the frequency of eYFP^{+} cells in each subset. Each symbol represents an individual animal and the horizontal bar represents the mean of n=7 mice from a single experiment. The lower histogram shows CD11c expression by DC subsets, F4/80^{+}MHCII^{+} macrophages and total CD45^{+} leukocytes from the small intestine of 6 week old CD11c-Cre.*Rosa26*-LSL-eYFP mice. (**b**) Representative expression of eYFP by CD19^{+} MHCII^{+} B cells and CD3^{+} MHCII^{-} T cells from the small intestine of CD11c-Cre.*Rosa26*-LSL-eYFP mice or CD11c-Cre mice (shaded histograms). Scatter plots show the frequency of eYFP^{+} cells in each lymphocyte subset. Each symbol represents an individual animal and the horizontal bar represents the mean n=7 mice from a single experiment. The lower histogram shows CD11c expression by CD19^{+} MHCII^{+} B cells, CD3^{+} MHCII^{-} T cells and total CD45^{+} leukocytes from the small intestine of CD11c-Cre.*Rosa26*-LSL-eYFP mice. (**c**) Representative expression of eYFP by neutrophils, classical Ly6C^{hi} monocytes, non-classical Ly6C^{lo} monocytes, CD19^{+} MHCII^{+} B cells and CD3^{+} MHCII^{-} T cells from the blood of CD11c-Cre.*Rosa26*-LSL-eYFP mice or CD11c-Cre mice (shaded histograms). Scatter plots show the frequency of eYFP^{+} cells in each leukocyte subset. Each symbol represents an individual animal and the horizontal bar represents the mean n=7 mice from a single experiment. The histogram (*right*) shows CD11c expression by each blood leukocyte subset from blood of CD11c-Cre.*Rosa26*-LSL-eYFP mice.

Supplementary Figure 4

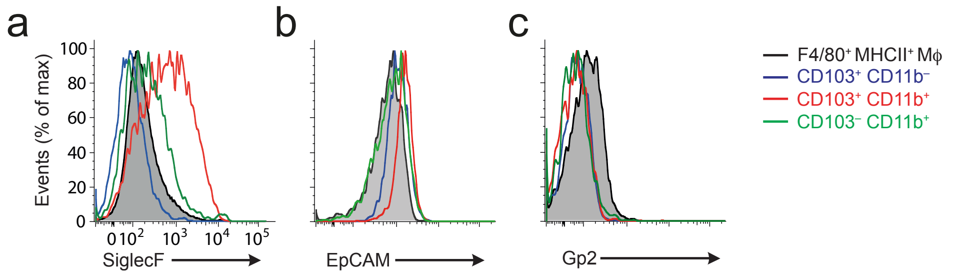

Fig. S4: Assessment of markers identified by microarray analysis at protein level

Representative expression of SiglecF (**a**), EpCAM (**b**) and GP2 (**c**) by CD103/CD11b-defined DC subsets and by F4/80$^+$MHCII$^+$ macrophages from the small intestinal LP of unmanipulated WT mice. Data are from a single experiment.

Supplementary Figure 5

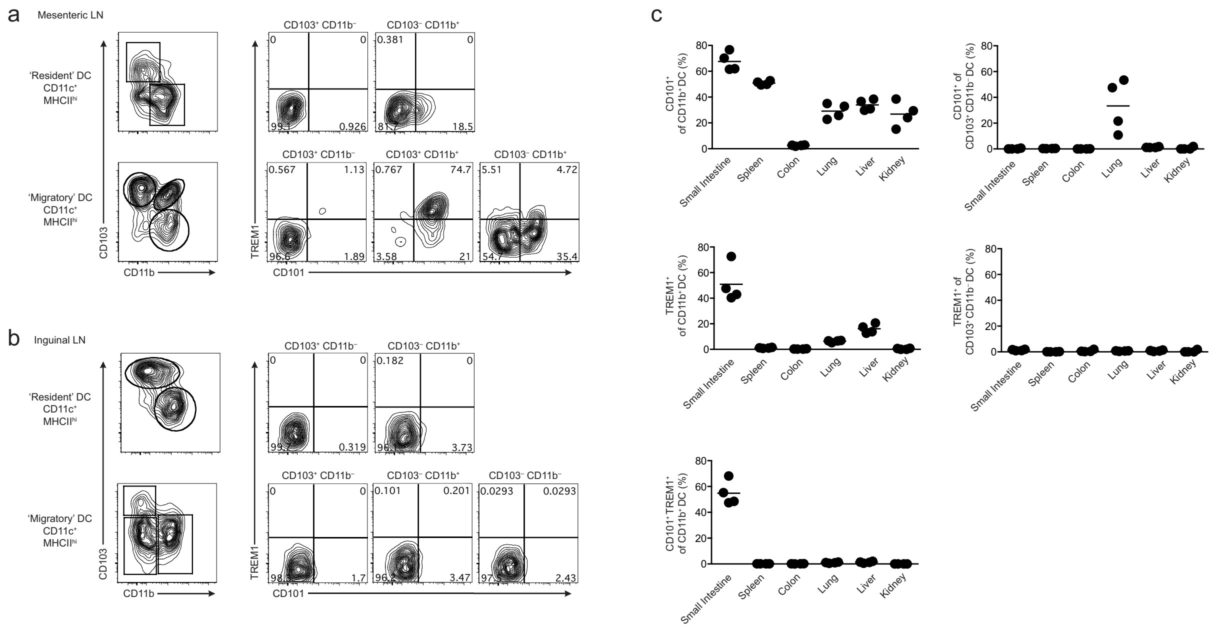

Fig. S5: Expression of CD101 and TREM1 by lymphoid and non-lymphoid DC subsets

(**a-b**) Representative expression of CD103 and CD11b by 'resident' (CD11c^{+} MHCII^{+}) and 'migratory' (CD11c^{+}MHCII^{hi}) DC in the mesenteric (**a**) or inguinal (**b**) lymph nodes (*left panels*), and representative expression of CD101 and TREM1 by CD103- and CD11b-defined DC subsets (*right panels*). (**c**) Scatter plots show the frequency of CD101^{+}, TREM1^{+} or CD101^{+}TREM1^{+} DC in the tissues indicated. Each symbol represents an individual animal and the horizontal bar represents the mean of 4 mice from one of two representative experiments performed.

Table S1: Genes unique to SILP CD103^{+}CD11b^{-} DC Subset

Clec9a	Tmem27	Clec1a	Sgcb	Slco3a1	Map2k6
Xcr1	Slc8b1	Pdlim7	Myo9a	Nab2	Lap3
A530099J19Rik	Alms1	Ptgis	Gng12	Glce	Ece1
Gpr33	Casp6	Fbxo4	Bcl2l14	Rab39	Arhgap21
5430435G22Rik	Fgd6	Itga3	Txndc15	Olfr433	Pdlim4
Ctla2b	Gnb4	Sdc3	Mllt3	Arsb	Rab43
Ffar4	Tlr11	Arrb1	Esyt3	Nup93	Rab13
Irf8	Fndc7	Pttg1ip	Extl3	Trim35	Cdon
Tlr12	Pbx1	Nek6	Sgk1	Galnt7	Bcl9
Ifi205	Dbn1	Gcsam	A630072M18Rik	Hk3	Lrrcc1
Cadm1	Clec12a	Dnase1l3	Cttnbp2nl	Sept3	Nampt
Tlr3	Trpm2	Gprc5c	Procr	Ndrg1	Pygo2
Ppap2a	Pdia5	Cyfip2	Naga	Gm6226	Pus3
Cxcr3	Arhgap42	Gatm	Cdk14	Rcn2	Rab11a
Cd8a	Cxcl9	Lrrc1	Laptm4b	Osbpl3	Klra5
Snx22	Arhgef9	Pstpip2	Il15	Rala	Tmtc3
P2ry14	Stk39	Serpinb8	Pfkfb1	Sdad1	Mgat4b
Cd36	Itm2a	Adam8	Tap1	Acadl	Gdpd5
Agpat3	Ppef2	Ubash3b	Tspan33	Slc31a2	Ptplb
F630111L10Rik	Itga1	Fzd1	Hps4	Rhobtb1	Ipcef1
Cd81	Ptger2	Cldn1	2610034B18Rik	Crim1	Adam19
Lif	Ccr9	Cpne3	Dhrs9	Psen2	Gpr157
Btla	Mras	Klf2	Slc9a9	Dpy19l3	Apol7c
Dapk2	Dusp10	Cdc14a	Pde1b	Pqlc2	Dnmbp
Ttc39a	Enpp4	Hmgn3	Tubb2b	Plin2	Fam168a
Leprel1	Fam149a	Arap1	Adrb2	Ubfd1	Ptpn18
Fnip2	Mpeg1	4930486L24Rik	Stt3b	Nck2	Gabbr1
Slc7a8	Phyh	Mpzl2	Chst11	Asap2	Myo6
Met	Rab32	Cpd	Slc33a1	Fndc9	Fmo5
Ppt1	Itga8	Fbxw17	Gna15	Mctp1	Mospd2
Naaa	Acvrl1	Cxx1c	2410016O06Rik	Aagab	Slc9a3r2
Cpq	Cd200	Oasl1	Tcf7l2	Agpat5	Rasgrp3
Mospd1	Erc1	Camk1d	Gusb	6820445E23Rik	Zc3h12c
Plce1	Gkap1	Fmnl2	Gm1965	Ccndbp1	Sult1a1
Pi4k2a	BC028528	Enpp5	Galc	Ppp1r11	Id2
Il12b	Igf2r	Clnk	Tubb2a	Zfp366	Gm11545
E330020D12Rik	B3gnt5	Ly75	Impa2	Sipa1l1	Zfp57
Cyp26b1	Mycl	Slc25a26	Tmem86a	Trim30a	Qpct
Zdhhc2	Slc25a24	Plcb4	Cyb5a	Coro7	Actn1
Lima1	Sgms1	Ldoc1l	Ankrd12	St3gal5	

Table S2: Genes shared by SILP CD103^{+}CD11b^{+} and CD103^{-}CD11b^{+} DC Subsets

Dhrs3	Tcn2	Pvr	Zfp667	Sorl1	Cd69
Apba1	Arl4c	Lrrc16a	Gp49a	Ehf	Clec10a
Il13ra1	Neurl3	Rasgrp2	Trps1	Oas1a	Ccrl2
Hfe	Gm23100	G6pdx	Il18	Ston2	Fgr
Arrb2	Pcyt1a	Chd7	Abcb1a	Tnfsf8	Cd72
Tmcc3	Rgs12	Tnfrsf18	Klk8	Kcne3	Clec4b1
Ncf1	Il21r	Arl5c	Tyrobp	Stk38l	Mmp12
Mreg	Bin1	Fam49a	E230008N13Rik	Spns3	Clec4a1
Ccdc88c	Stard9	Capg	Siglecg	Ffar2	Ctnnd2
Rnf149	Chn2	Rgs1	Ifitm6	Atrnl1	Pygl
Stap1	Ssh2	Ltb4r1	Lilrb4	Hilpda	Tgm2
Isg20	Plcxd2	Acsl1	Tnip3	Fam46a	Irf4
Fmnl3	Plxnc1	Etos1	Atp13a3	Fosl2	B4galt6
Cebpa	Ly86	1700064E03Rik	Gch1	Abcg3	Stat4
Cyp4f16	Dusp22	Ppp1r3b	S100a10	Rgl1	Slc15a3
LOC637260	Zcwpw1	Stambpl1	Cass4	Sema7a	Cd300a
Micu1	Dock4	Ank	Ndst1	Ms4a6d	Clec4n
Cd164	Csf2rb	Syk	Trp53i11	Gpr141	Clec4a2
Ldlr	Ifi27	Bach1	AW112010	Nod1	Cd209b
Mxd1	Cdkn1a	Pou2f2	Ddx58	Frrs1	Ptgs2
Fhl3	Prkch	Ntpcr	Zeb2	Malt1	Sh2d1b1
Ms4a6c	Sidt2	Slc2a6	Slc2a3	Fgfr1	Cd7
Tmem106a	Elmsan1	Rasal3	Oasl2	Gbp3	Clec4a4
Pde2a	Panx1	I730030J21Rik	Cyp4f18	Fabp5l2	Emb
Smpdl3b	Map4k3	Arhgap39	Cd300ld	Gbp4	Fcer1g
Por	Gpr183	Slc16a3	Nfkbiz	Ramp3	Cd209c
Casp1	Lgals8	Emilin2	A530064D06Rik	Art2a-ps	Atp1a3
Gm14455	Hrh1	Fyb	Ggt5	Clec4g	Pltp
Cxcr4	Ripk2	Lst1	Acp5	Lair1	Pilra
Nrros	Irak2	Ggta1	Gadd45b	Gcnt2	Il1r2
Ctsz	Tpp1	Tnfrsf1b	Gm9733	Arhgef3	Cybb
Napsa	Plxnd1	Tbx21	I830077J02Rik	Milr1	Trem1
Dusp7	Tfrc	Zeb2os	Gm5150	Nlrp3	Sirpa
Igkv3-9	Pla2g4a	Lgals9	Arap2	Rtp4	Ccr1
Il1rl2	Rab20	App	Gm1966	Pecam1	Cd33
Stk17b	Pirb	Ddx26b	Ncf2	Emr1	Mgl2
Adora2b	Hcar2	Pla2g7	Cd274	Mefv	Siglece
Spib	Nfkbid	Ccnd2	Wls	Pilrb2	Itgam
Crtc3	Cers6	Igsf6	Lifr	F11r	1810011H11Rik
Trappc5	Smox	Dgat1	Ifitm1	1300002K09Rik	Cd209a
Sulf2	AF251705	Mycbp2	Samsn1	Il1b	Cd101
Abi3	Ctsa	Zfp719	Rin2	Prdm1	Wfdc17
Ifi27l2a	Lpp	Ctsc	Ifitm2	Cd300lf	Ccl3
Rbpms	Tctex1d2	Nedd9	B430306N03Rik	Plaur	Serpinb10
Dhx40	Cd14	Klrd1	Fcgr3	Plau	Dnajb4
Krt80	Pilrb1	Cfp	St18	Clec7a	Tlr1
Lta4h	Casp4	Ddr1	Bhlhe40	Gbp8	Oas2
Gngt2	St6gal1	Gapt	Ifit3	Mcemp1	Ltb
Zc3hav1	Pvt1	Mmd	Mgat4a	Hpgd	Lat2
Mvb12b	Ptger4	Nr4a3	Plk2	Il1a	Hivep2
Smad7	Vopp1	Tlr2	Dennd3	Lphn3	Nxpe5
Neurl2	Trem3	Ggh	Ms4a6b	Gbp7	Bcl3
Igkv4-57	Lpcat2	Ier2	Arid5a	Maml3	Ddx60

Il7r	Dusp3	Rsad2	Bcl11a	Coro2a	Elk3
Gm8995	Slc44a2	St7	Eif4e3	Runx3	Lamp1

Table S3: Genes unique to the SILP CD103$^-$CD11b$^+$ DC Subset

mem176b	Il17ra
Tnfsf9	Lilra6
Tmem176a	Msr1
Apobec1	Ccl9
Csf1r	Il1rn
C1qb	Nccrp1
C1qc	Abcb1b
S100a4	St8sia6
C1qa	Plxnb2
Dab2	Slamf9
Klra17	Ccl4
Pla2g2d	Cx3cr1
S100a6	Mmp9
Ccl6	C3ar1
Ptafr	Alox5
Plxdc2	Mef2c
Osm	Prkce
Ms4a4c	Dram1
Il22ra2	Lpar1
Emr4	BC005685
Apoe	Rasgrp1
Klra2	St8sia1
Mrc1	Anxa3
Emp3	Rasgef1b
Clec4a3	Clec5a
Mafb	Cd93
Fcgr2b	Rassf4
Csf3r	Cercam
Abca9	Spn
Socs3	Foxred2
Mzb1	

Table S4: Genes unique to the SILP CD103$^+$CD11b$^+$ DC Subset

Impact	Ppp1r14a
Slc27a2	Rasgrf2
Gcnt1	Card9
Pglyrp1	Pgf
Epcam	Ppp1r1a
Slc16a6	Mospd4
Rilpl1	Tnfaip2
Abhd14b	Mtss1
Mylk	Trim25
2900026A02Rik	Siglec5
Rell1	Fam189a2
Sash1	Cdh1
D330045A20Rik	Cpm
Cwc25	Plet1
Plk3	Gp2
Slc52a3	

Table S5: Genes shared by SILP CD103^{+}CD11b^{+} and CD103^{-}CD11b^{+} DC subsets and highly significantly differentially expressed compared with CD103^{+}CD11b^{-} DC.

Gene	Surface marker	FACS Antibody
Plaur	x	
Mcemp1	x	
Clec10a	x	
Clec4a1		
Tgm2		
Irf4		
B4galt6		
Stat4		
Slc15a3		
Cd300a	x	x
Clec4n	x	
Clec4a2	x	
Ptgs2		
Sh2d1b1		
Cd7	x	
Clec4a4		
Emb	x	
Fcer1g	x	
Cd209c		
Atp1a3		
Pltp	x	
Pilra	x	
Il1r2		
Cybb		
Trem1	x	x
Sirpa	x	x
Ccr1		
Cd33		
Mgl2	x	x
Siglece	x	x
Itgam	x	x
1810011H11Rik		
Cd209a	x	x
Cd101	x	x
Wfdc17		

Table S6: Antibodies used for Flow Cytometry

Antibody	Clone	Source	Dilution
CD3e	145-2c11	Biolegend	1:200
CD4	RM4-5	Biolegend	1:200
CD11b	M1/70	Biolegend	1:200
CD11c	N418	Biolegend	1:200
CD44	IM7	Biolegend	1:200
CD45	30-F11	Biolegend	1:200
CD45.1	A20	Biolegend	1:200
CD45.2	104	Biolegend	1:200
CD62L	MEL-14	Biolegend	1:200
CD64	X54-5/7.1	Biolegend	1:200
CD69	H1.2F3	Biolegend	1:200
CD101	Moushi101	eBioscience	1:200
CD103	2E7	Biolegend	1:200
EpCAM	G8.8	Biolegend	1:200
FoxP3	FJK16s	eBioscience	1:200
GP2	D278-A48	MBL	1:200
IA-IE	M5/114.15.2	Biolegend	1:400
IFNγ	XMG1.2	BD Bioscience	1:200
IL17A	TC11-18H10.1	Biolegend	1:200
Ly6C	HK1.4	Biolegend	1:200
SiglecF	E50-2440	BD Bioscience	1:200
TREM1	TR3MBL1	eBioscience	1:200
Vα2	B20.1	Biolegend	1:200
Fc Block	2.4G2	Biolegend	1:200

Table S7: Primers used for Genomic PCR

Gene	Primer sequences	Product size (in Kb)
Tgfbr1	5’- ACCCTCTCACTCTTCCTGAGT- 3’<br>5’- ATGAGTTATTAGAAGTTGTTT- 3’<br>5’- GGAACTGGGAAAGGAGATAAC- 3’	WT = 150<br>Flox = 250<br>Del = 350
Cre	5’- CCGGTCGATGCAACGAGTGA- 3’<br>5’- GGCCCAAATGTTGCTGGATA- 3’	WT = absent<br>Cre = 235
Rag1	5’ – TGGATGTGGAATGTGTGCGAG- 3’<br>5’ – GAGGTTCCGCTACGACTCTG- 3’<br>5’ – CCGGACAAGTTTTTCATCGT- 3’	WT = 474<br>Del = 520

Table S8: Primers used for RT-qPCR

Gene	Forward primer sequence	Reverse primer sequence
Il6	TCTAATTCATATCTTCAACCAAGAGG	TGGTCCTTAGCCACTCCTTC
Il1b	TGTAATGAAAGACGGCACACC	TCTTCTTTGGGTATTGCTTGG
Tnfa	CCACGTCGTAGCAAACCAC	TTTGAGATCCATGCCGTTG
Ifng	ATCTGGAGGAACTGGCAAAA	TTCAAGACTTCAAAGAGTCTGAGGTA
Il17a	TTTTCAGCAAGGAATGTGGA	TTCATTGTGGAGGGCAGAC
Nos2	GGGCTGTCACGGAGATCA	CCATGATGGTCACATTCTGC
Ccl5	TGCAGAGGACTCTGAGACAGC	GAGTGGTGTCCGAGCCATA
Gzmb	GCTGCTCACTGTGAAGGAAGT	TGGGGAATGCATTTTACCAT
